# Physiological and transcriptomic responses of water spinach (*Ipomoea aquatica*) to prolonged heat stress

**DOI:** 10.1186/s12864-020-06953-9

**Published:** 2020-08-03

**Authors:** Rongfang Guo, Xingru Wang, Xiaoyun Han, Xiaodong Chen, Gefu Wang-Pruski

**Affiliations:** 1 Joint FAFU-Dalhousie Lab, College of Horticulture, Fujian Agriculture and Forestry University, Fuzhou, 350002 China; 2 College of Horticulture, Fujian Agriculture and Forestry University, Fuzhou, 350002 China; 3 Department of Plant, Food, and Environmental Sciences, Faculty of Agriculture, Dalhousie University, Truro, NS B2N 5E3 Canada

**Keywords:** *Ipomoea aquatica*, Long-term heat stress, RNA-seq, Thermotolerance, Starch and sucrose metabolism

## Abstract

**Background:**

Water spinach (*Ipomoea aquatica*) is an important heat-resistant leafy vegetable that can survive under long-time heat stress condition. However, the physiological characteristics and molecular changes in its response to heat stress are poorly understood.

**Results:**

In this study the selected water spinach cultivars with different thermo resistance and their physiological response to heat stress were examined. Under prolonged heat stress, plant growth was inhibited in all tested cultivars. This inhibition was accompanied by the reduction of photosynthetic performance. The reactive oxygen species system in terms of superoxide and hydrogen peroxide contents, as well as antioxidant polyphenols, were evaluated. The results showed that prolonged heat stress caused reduced antioxidant capacity, but the role of antioxidant capacity in a prolonged thermotolerance was not predominant. Transcriptomic analysis of the water spinach subjected to heat stress revealed that 4145 transcripts were specifically expressed with 2420 up-regulated and 1725 down-regulated in heat-sensitive and heat-tolerant cultivars treated with 42 °C for 15 days. Enrichment analysis of these differentially expressed genes showed that the main metabolic differences between heat-sensitive and heat-tolerant cultivars were the carbohydrate metabolism and phenylpropanoid biosynthesis. The results of carbohydrate profiles and RT-qPCR also suggested that heat stress altered carbohydrate metabolism and associated changes in transcriptional level of genes involved in sugar transport and metabolic transition.

**Conclusions:**

The prolonged heat stress resulted in a reduced antioxidant capacity while the role of antioxidant capacity in a prolonged thermotolerance of water spinach was not predominant. Transcriptome analysis and the measurement of carbohydrates as well as the gene expression evaluation indicated that the response of the metabolic pathway such as carbohydrate and phenylpropanoid biosynthesis to heat stress may be a key player in thermo resistance.

## Background

Water spinach (*Ipomoea aquatica*), which belongs to the Convolvulaceae family, is an important aquatic or semi-aquatic vegetable that naturally grows in Asia and southwestern Pacific islands during summer and autumn [1]. Water spinach, originated from tropical regions, is consumed as a leafy vegetable with high tolerance to heat and wet [2, 3]. The plant contains high level of nutrients, such as essential amino acids, vitamin A, vitamin C, and iron, as well as polyphenols including carotenoids and chlorophylls [1].

Atmosphere temperature has increased since the beginning of the last century and it is predicted to further increase under the present climate change [4]. The increasing temperature poses an imminent challenge to plant growth, development, and yield across the world [5]. Heat stress occurs when plants exposed to temperatures above the optimal for growth for some time. Under heat stress condition, extensive protein denatures and aggregates and the membrane system’s integrity and fluidity would be damaged [6]. Thermotolerance refers to the ability of an organism to cope with excessive high temperatures by physiological and biochemical adaptations [7]. The alterations of enzyme activity are also accompanied with the changes in temperature which may cause the imbalance of metabolic processes. During thermotolerance, reactive oxygen species (ROS) were produced and accumulated in different organisms which would cause the oxidative stress in plants. It has been reported that heat tolerant plants have stronger scavenging ROS genes compared with heat sensitive ones [8] and that enhancing the activity of antioxidant enzymes could improve the tolerance of plant to heat stress [9]. However, the molecular events occurring in response to heat stress have been mainly studied in experimental plants shortly after a brief exposure to high temperature, whereas research about the effects of prolonged high temperature exposures in plants is still limited [10–12]. The mechanism underlying short-term heat response and chronic thermotolerance may be different. The transcription factors related to growth and development were repressed by long-term heat stress in swichgrass (*Panicum virgatum*) [12] while a large amount of transcription factors were induced by short-term heat stress in wheat (*Triticum aestivum*) [13]. The common genes identified in short-term and long-term heat stress are related with protein folding and unfolding [12–14].

RNA sequencing (RNA Seq) is an effective and widely used technique for exploring genes associated with heat resistance, especially for these plants which genome sequences are not available. Li et al. (2013) identified 2000 up-regulated and 2809 down-regulated unigenes in switchgrass under long-term heat stress treatment (38 °C/30 °C, day/night, for 50 days) by transcriptome analysis [12]. Gonzálezschain et al. (2015) revealed molecular basis of heat stress responses in tolerant and sensitive rice varieties during anthesis by RNA Seq [15]. Gao et al. (2017) characterized the digital gene expression signatures of *Clematis apiifolia* under heat-stress conditions by transcriptome profiling [16]. Water spinach, an excellent heat-resistant species can provide important materials for investigating the molecular mechanisms of long-term heat stress [17]. The current study intended to assess the influence of long-term heat stress on the photosynthesis and antioxidant capacity of water spinach, and to identify genes and regulatory networks involved in long-term thermotolerance by transcriptome sequencing of heat-tolerant and heat-sensitive water spinach cultivars. This study will help us to improve the understanding of the mechanisms of the physiological and molecular changes of water spinach to heat stress and facilitate increasing heat tolerance in plants.

## Results

### Effects of the heat stress on morphological characterization and photosynthetic pigment contents in water spinach

**Fig. 1:**
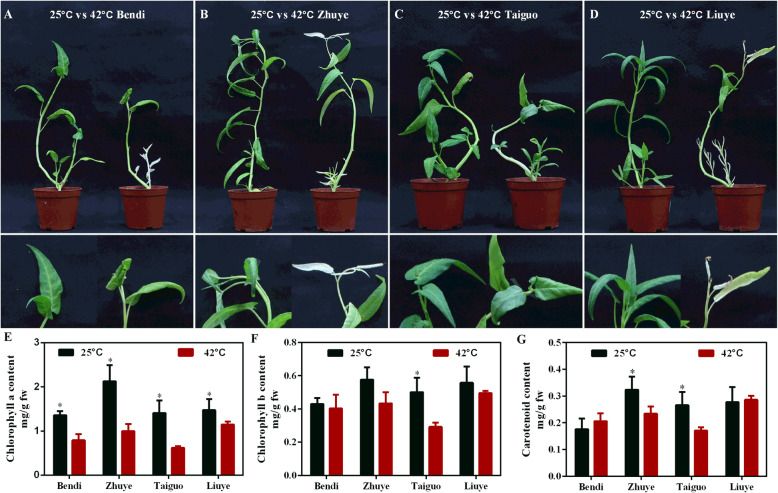
Morphological characterization and photosynthetic pigment contents of four water spinach cultivars cultured at 25 °C and 42 °C for 15 days, respectively. The four cultivars are ‘Bendi’ (**a**), ‘Zhuye’ (**b**), ‘Taiguo’ (**c**), and ‘Liuye’ (**d**). Upper panel: the whole water spinach plant of the four varieties subjected to 25 °C and 42 °C for 15 days, respectively. Lower panel: the enlarged (2X) apical bud of the corresponding water spinach plant in the upper panel. The contents of photosynthetic pigments include chlorophyll a (**e**), chlorophyll b (**f**), and carotenoid (**g**) of the four cultivars at 25 °C and 42 °C for 15 days, respectively. The statistical analyses were conducted in the same variety under different temperature conditions and the significant one was labelled with asterisks (*)

To investigate the thermo-resistant mechanism of water spinach, four different water spinach cultivars (‘Bendi’, ‘Liuye’, ‘Taiguo’ and ‘Zhuye’) were grown at 42 °C and 25 °C (control) for 15 days. As shown in Fig. 1a-d, irregular white spots appeared on lower leaves in these water spinach cultivars under heat stress compared to the cultivars cultured at 25 °C, especially the ‘Liuye’ cultivar showed the most albinism (Fig. 1d), while ‘Taiguo’ cultivar was the least (Fig. 1c). The branch leaves of cvs ‘Bendi’ and ‘Zhuye’ were yellow, the apical bud on main stem of cv. ‘Bendi’ is still green (Fig. 1a) and functional while it is dying in cv. ‘Zhuye’ (Fig. 1b). The contents of photosynthetic pigments including chlorophyll a, chlorophyll b and carotenoid of these cultivars under heat stress condition were tested (Fig. 1e-g). Compared with the cultivars cultured at 25 °C, the content of chlorophyll a (Fig. 1e) in all cultivars decreased after heat exposure. The accumulation of chlorophyll b (Fig. 1f) in cv. ‘Taiguo’ and carotenoid (Fig. 1g) in cvs ‘Zhuye’ and ‘Taiguo’ was also reduced after plants were treated with 42 °C for 15 days. Collectively, plant vegetative growth was inhibited and photosynthetic pigment contents decreased under long-term heat stress.

### Effects of heat stress on photosystem in water spinach

**Fig. 2:**
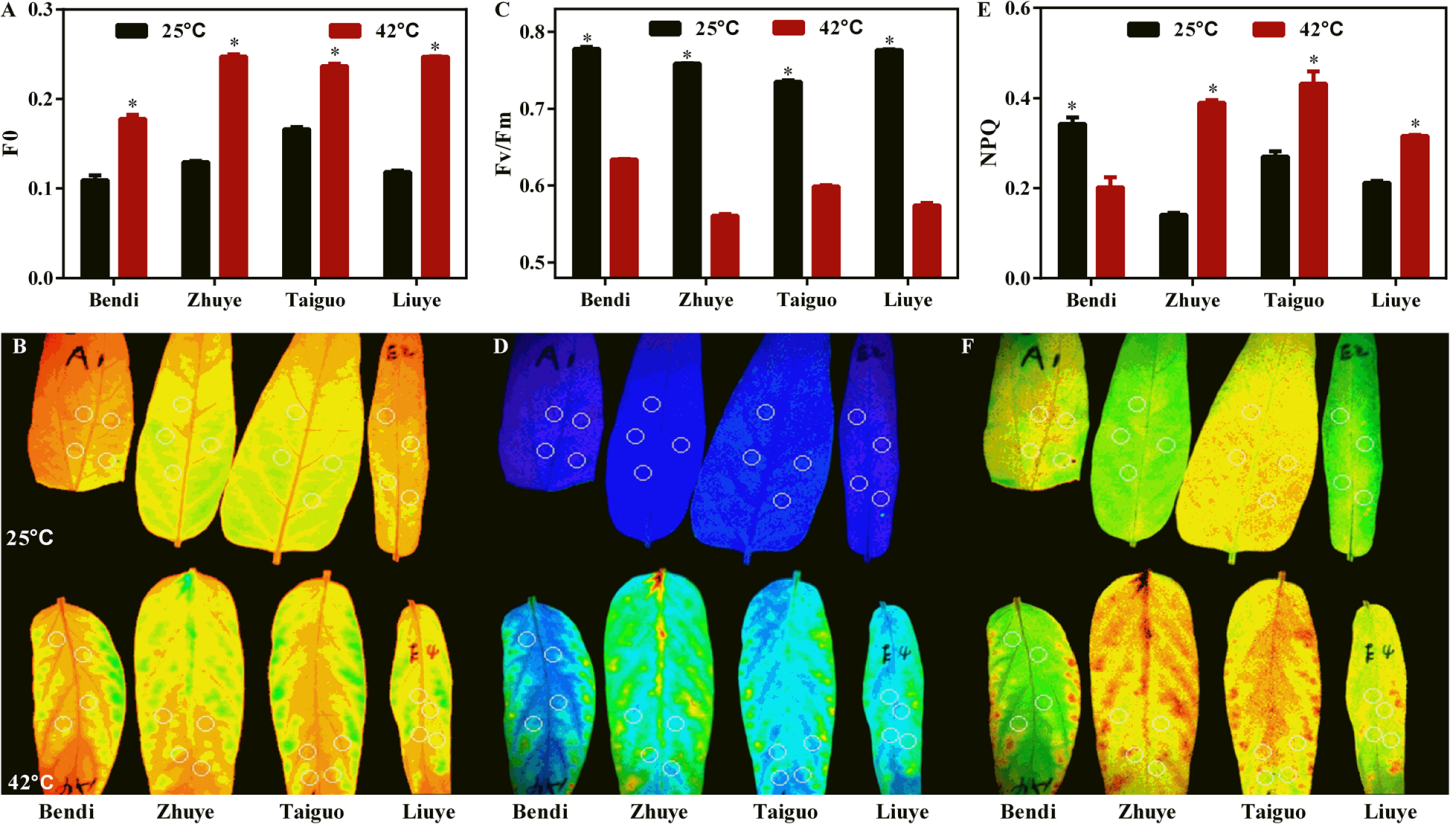
Effects of the heat shock on some characteristics related to photosynthetic system in water spinach. **a** Initial fluorescence value (F_0_) and (**b**) Color visual images of dark-adapted leaves in four cultivars at 25 °C and 42 °C, respectively. **c** Quantum efficiency of open PSII reaction centers in the dark-adapted state (Fv/Fm) and (**d**) color visual images of dark-adapted leaves in four cultivars at 25 °C and 42 °C, respectively. **e** Non-photochemical fluorescence quenching (NPQ), and (**f**) color visual images of dark-adapted leaves in four cultivars at 25 °C and 42 °C, respectively. Every column in each graph represents the mean (± S.E.) of three replicates. The statistical analyses were conducted in the same variety under different temperature conditions and the significant one was labelled with asterisks (*)

Leaf photosynthesis is one of the most heat-sensitive physiological processes [18]. Since the results above showed the reduction of photosynthetic pigment contents in water spinach under heat stress, we evaluated the effects of heat stress on photosynthetic performance in different cultivars (Fig. 2). The results showed that the initial fluorescence level from dark-adapted leaves (F_0_) was increased in all cultivars after heat exposure (Fig. 2a, b), while maximum quantum efficiency of photosystem II (PSII) photochemistry (Fv/Fm) was decreased (Fig. 2c, d). These results suggested that prolonged heat stress harms PSII reaction centers. The effects of heat stress on photosynthetic performance were further evaluated by analyzing chlorophyll fluorescence quenching. There was a significant reduction of non-photochemical fluorescence quenching (NPQ) value, the most efficient photoprotective response to dissipate as heat in order to prevent ROS production in plants, in cv. ‘Bendi’ under heat stress condition, whereas the NPQ values in cvs ‘Zhuye’, ‘Taiguo’, and ‘Liuye’ were elevated (Fig. 2e, f). These results suggested that photosynthetic efficiency decreased under long-term heat stress and the effect of heat stress on leaf photosynthesis differ between genotypes.

### Effects of the heat stress on antioxidant capacity in water spinach

**Fig. 3:**
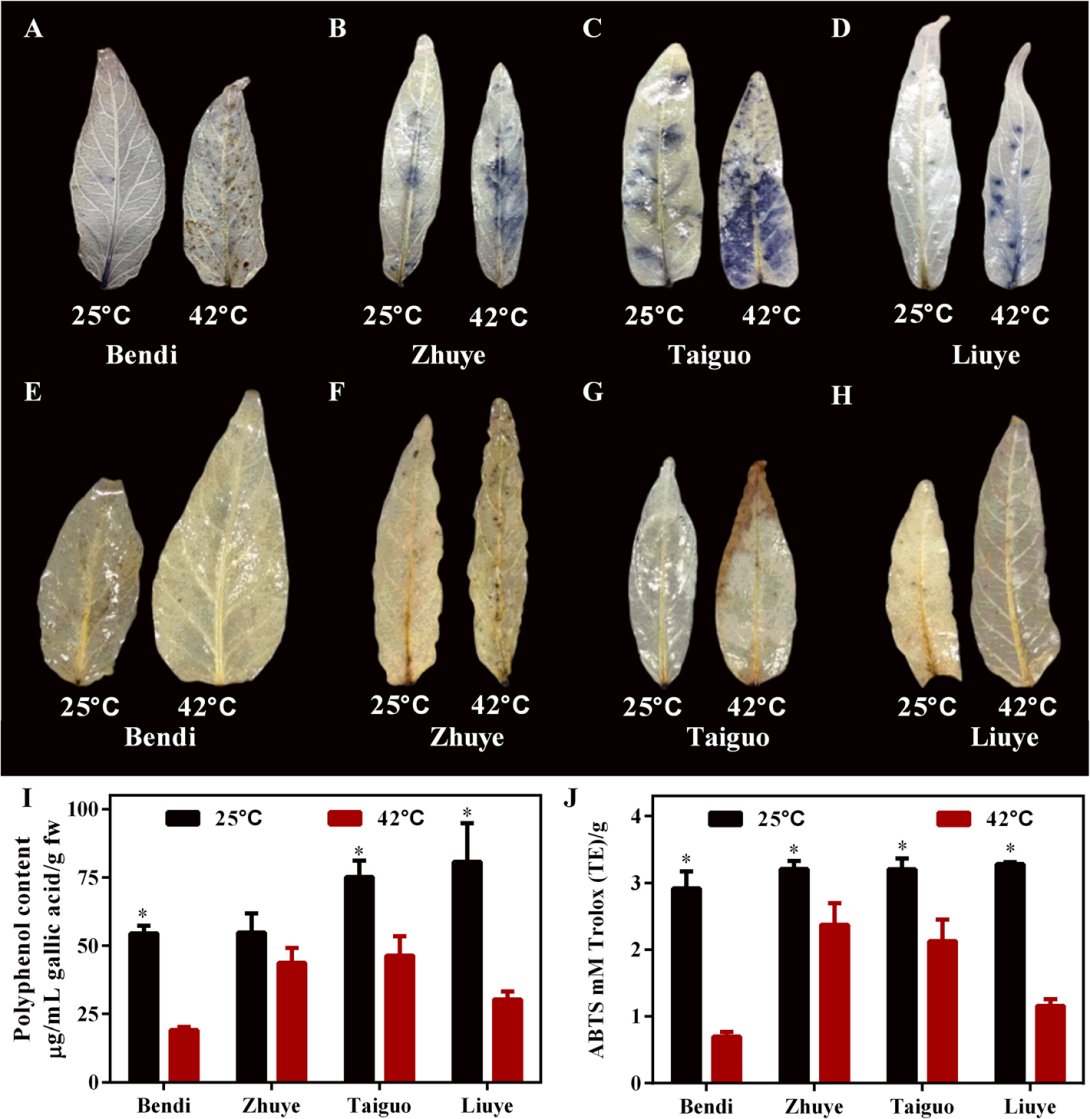
Effect of the heat shock on superoxide (O_2_·^−^), hydrogen peroxide (H_2_O_2_) content and antioxidant ability in four water spinach cultivars cultured at 25 °C and 42 °C for 15 days, respectively. **a-d** The generation of O_2_·^−^ in water spinach leaves measured by nitro blue tetrazolium (NBT) reduction method in four cultivars, respectively. **e-h** The H_2_O_2_ content of water spinach measured by 3, 3′-diaminobenzidine (DAB) method in four cultivars, respectively. **i** The polyphenol content of leaves in four water spinach cultivars cultured at 25 °C and 42 °C, respectively. **j** The total antioxidant capacity measured by 2, 2-azobis-3-ethylbenzothiazoline-6-sulfonic acid (ABTS) in four water spinach cultivars cultured at 25 °C and 42 °C, respectively. The statistical analyses were conducted in the same variety under different temperature conditions and the significant one was labelled with asterisks (*)

The generation of ROS and ROS-scavenging capacity in leaves of four cultivars were determined after exposure to high temperature. The generation of O_2_·^−^ in water spinach leaves was detected using nitroblue tetrazolium (NBT) reduction method. As shown in Fig. 3a-d, blue precipitate representing O_2_·^−^ content was observed in four cultivars exposure to heat stress. Consistently with the change of NPQ value (Fig. 2c), the least blue dots were found in cv. ‘Bendi’ (Fig. 3a). Under heat stress condition, the production of O_2_·^−^ was increased in heat-sensitive cv. ‘Zhuye’ (Fig. 3b) and cv. ‘Liuye’ (Fig. 3d), although increased content of O_2_·^−^ was also detected in heat-resistant cv. ‘Taiguo’ (Fig. 3c). It should be noted that leaves of cvs ‘Zhuye’ and ‘Taiguo’ under optimal growth condition also exhibited a large production of O_2_·^−^, indicating the ROS accumulation varied among varieties. The effect of heat stress on H_2_O_2_ content analyzed by 3, 3′-diaminobenzidine (DAB) method was shown in Fig. 3e-h, where the H_2_O_2_ accumulation was found the most in a heat-sensitive cv. ‘Zhuye’ and the least in cv. ‘Bendi’. However, more precipitates were observed in heat-resistant cv. ‘Taiguo’ than in cv. ‘Liuye’, indicating that the accumulation pattern of H_2_O_2_ in water spinach was independent of their heat resistance characteristics.

To further detect the role of antioxidant ability in heat resistance, the antioxidant capacity in terms of polyphenol content and total antioxidant capacity of water spinach in response to heat stress was evaluated (Fig. 3i, j). The polyphenol content of leaves was significantly decreased by heat stress compared to the control, notably a sharp decline in cvs ‘Bendi’, ‘Taiguo’, and ‘Liuye’ while no obvious change in cv. ‘Zhuye’ (Fig. 3i). The same pattern of variation was observed in total antioxidant capacity of leaves in four cultivars after treatment with heat stress (Fig. 3j). After comprehensive analysis of O_2_·^−^ and H_2_O_2_ accumulation and antioxidant ability in heat-resistant cultivars ‘Bendi’ and ‘Taiguo’ as well as heat-sensitive cultivars ‘Zhuye’ and ‘Liuye’, it is proposed that no direct relationship exists between the heat resistance and antioxidant content in water spinach*.*

### RNA Seq and reads assembling of water spinach cultivars with different resistance to heat

After the phenotype analysis under heat stress condition, the ability of heat resistance from high to low is cvs ‘Taiguo’, ‘Bendi’, ‘Zhuye’, and ‘Liuye’. The first one cv. ‘Taiguo’ (T01) with the strongest resistance to heat and the last one cv. ‘Liuye’ (T02) with the weakest tolerance to heat were selected to perform RNA seq to further investigate the thermo-resistant mechanism of water spinach**.** Transcriptome sequencing yielded a total of 5,978,228,686 (T01) and 6,602,182,028 (T02) clean bases, and 23,750,150 (T01) and 26,225,685 (T02) clean paired-end reads after filtering out low-quality reads and trimming adaptor sequences (Table S1). After sequenced reads assembly by Trinity software, the mapped reads were 20,411,655 and 22,762,781, and mapped ratio were 85.94 and 86.8% in T01 and T02, respectively. The mapped reads were used for subsequent analysis. The GC percentage values were 46.79 and 46.55% in T01 and T02, respectively. The Q30 percentage (proportion of nucleotides with quality value larger than 30 in reads) values were 85.38 and 85.48% in T01 and T02, respectively (Table S1). To elucidate gene structures, single nucleotide polymorphisms (SNPs) were predicted. In this study, we identified and characterized SNPs in the genic and intergenic regions using SAM tools software. There was a total of 40,063 and 38,836 SNPs in T01 and T02, respectively. More heterogenetic SNPs in T01 while more homogenetic SNPs in T02 (Table S2).

### Gene expression comparisons in heat tolerant and sensitive water spinach cultivars

**Fig. 4:**
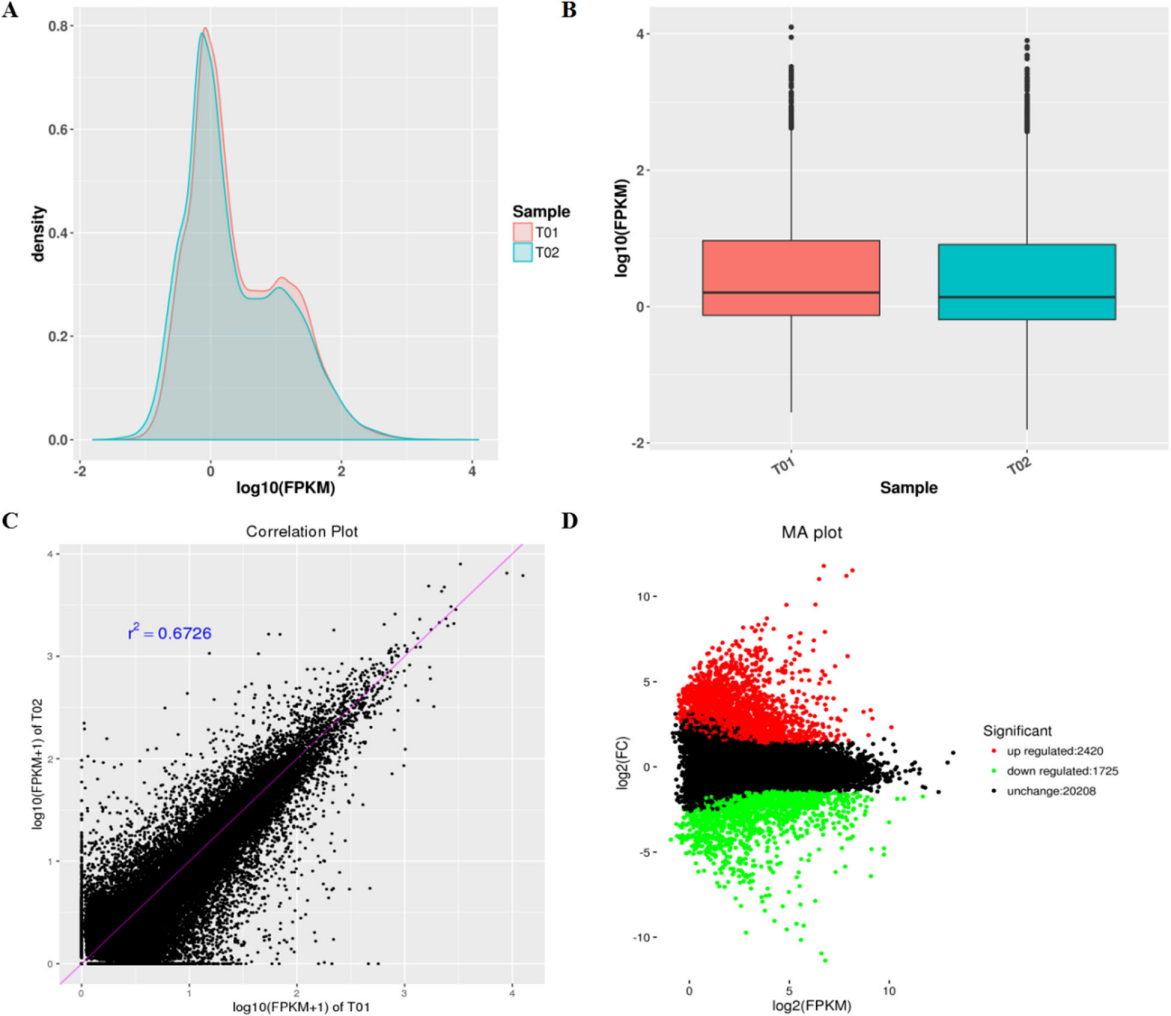
Gene expression comparisons of heat tolerant and sensitive water spinach cultivars. **a** FPKM density distribution of T01 and T02. The y-axis corresponds to gene density, and the x-axis displays the log_10_ (FPKM) of samples. **b** The boxplot of overall expression level of T01 and T02. **c** The scatter diagram of gene expression T01 and T02, x- and y-axis display the log_10_ (FPKM+ 1) of T01 and T02, respectively. **d** The MA plot presentation, the red dots represent up-regulated DEGs, the green dots represent down-regulated DEGs, and the black dots represent non-DEGs

Next, the gene expression levels of heat tolerant cv. ‘Taiguo’ (T01) and heat sensitive cv. ‘Liuye’ (T02) were analyzed. The gene expression levels based on FPKM and gene densities of T01 and T02 was shown in Fig. 4a, and the expression levels were delineated by boxplot profiles in Fig. 4b, which demonstrated a dispersion degree of gene expression in two samples. Then Pearson’s correlation coefficient (r^2^) of two sample was obtained as 0.6726, and more dots that deviated from the diagonal in the scatter diagram of gene expression were shown in Fig. 4c. These results suggested a great difference of gene expression level between T01 and T02. Based on FPKM values, 4145 differentially expressed genes (DEGs, with *p* < 0.005 and |log_2_ (fold change) | > 1) were identified. Among them, 2420 genes displayed up-regulation and 1725 genes displayed down-regulation in T02 vs T01 (Fig. 4d).

### Functional analysis and classifications of DEGs

**Fig. 5:**
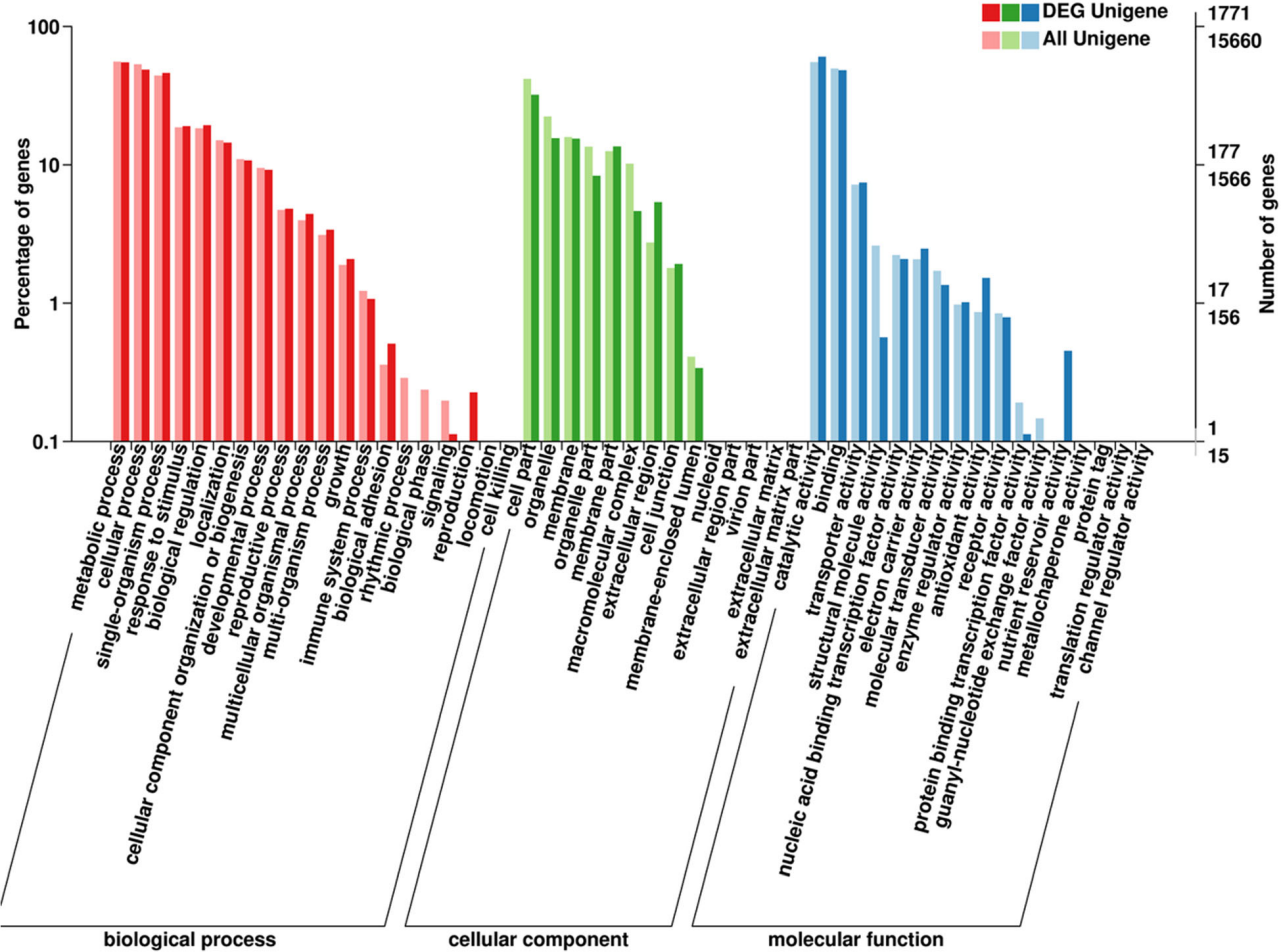
GO annotation of differentially expressed genes (DEGs) in T01 vs T02. GO analysis was performed for the three main categories (biological process, cellular component and molecular function). The X axis represented different categories of all Unigenes and differentially expressed Unigenes in T01 and T02. The left Y axis indicated the percentage of a specific category of all Unigenes and differentially expressed Unigenes in a specific category. The right Y axis indicated the numbers of all Unigenes and differentially expressed Unigenes in a specific category. Dark color columns are for DEG Unigenes; light color columns are for all Unigenes

To further study the function of DEGs, GO database was adopted to analyze the DEG functional classifications and the KEGG pathway database was initiated to identify the biological pathways of DEGs. The DEGs were divided based on biological process (BP), cellular component (CC), and molecular function (MF). The largest BP, CC, and MF subcategory for the DEGs in T02 vs T01 were oxidation-reduction process, integral component of membrane and ATP binding, which comprised 15 .47, 24.21 and 16.21% of the DEGs in the subcategory, respectively (Fig. 5).

**Fig. 6:**
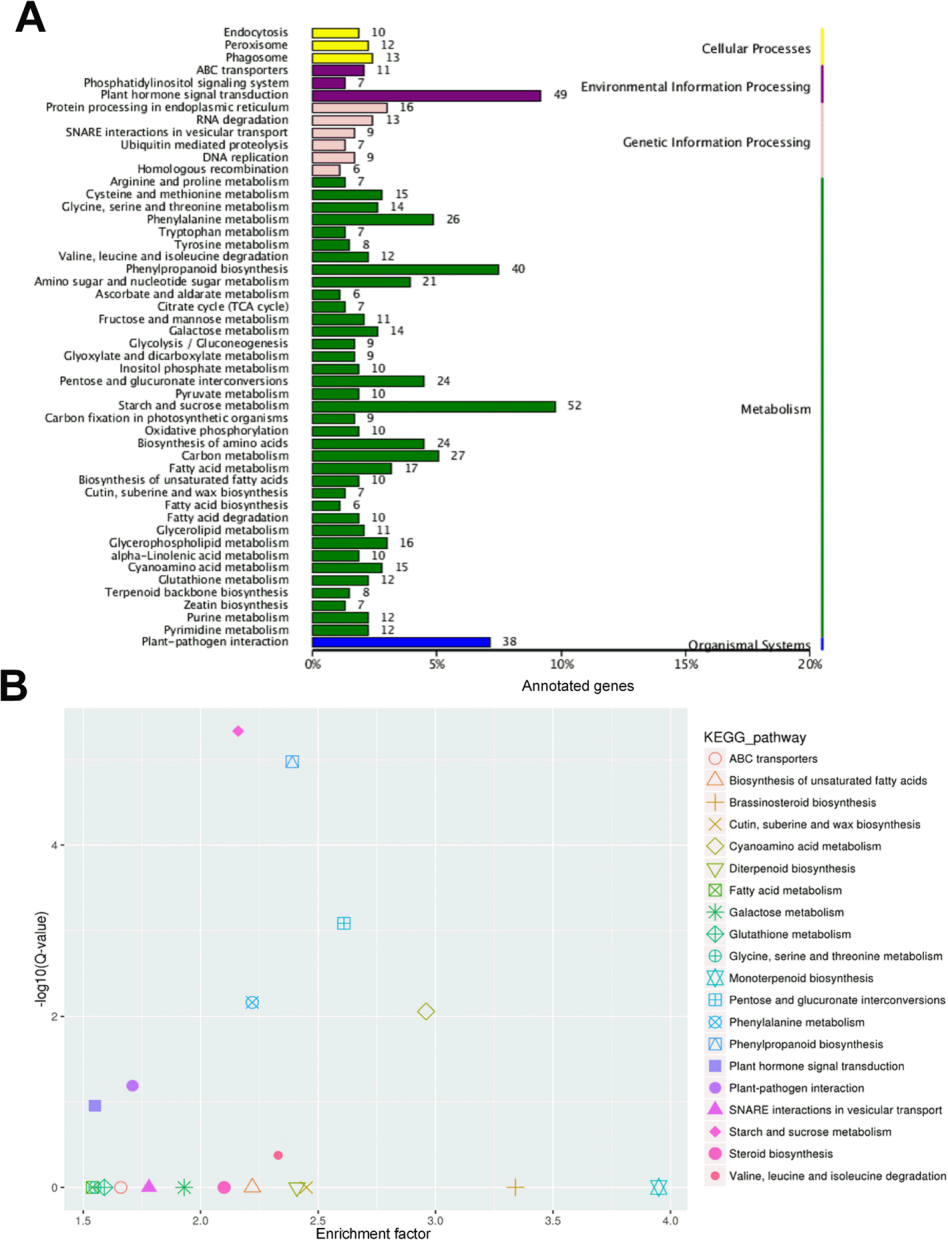
**a** KEGG annotation of DEGs in T01 vs T02. The X axis was the rate of annotated genes to different categories of KEGG. The Y axis represented different categories of KEGG in T01 and T02. Blue column, organismal systems; green column, metabolism; pink column, genetic information processing; purple column, environmental information processing; yellow column, cellular process. **b** KEGG enrichment of DEGs in T01 vs T02. The y-axis corresponded to different Q-value of different KEGG pathway, and the x-axis showed the enrichment factor of different KEGG pathway. The different symbols represented for different KEGG pathway

Using the KEGG pathway database, 114 biological pathways were identified. The top three most enriched KEGG pathway in T02 vs T01 were starch and sucrose metabolism, plant hormone signal transduction and phenylpropanoid biosynthesis, respectively (Fig. 6a). These changes indicate significant changes in the secondary metabolism in T02 vs T01 in response to long-term heat stress. Furthermore, the two top-ranking pathways in the significant enriched 20 KEGG pathways of T02 vs T01 were starch and sucrose metabolism and phenylpropanoid biosynthesis (Fig. 6b), which coincided with the analysis in Fig. 6a. Based on the analysis of transcriptome data, DEGs related to carbohydrate and phenylpropanoid were selected (Table S3 & S4).

### Carbohydrate profiles during long-term heat stress

**Fig. 7:**
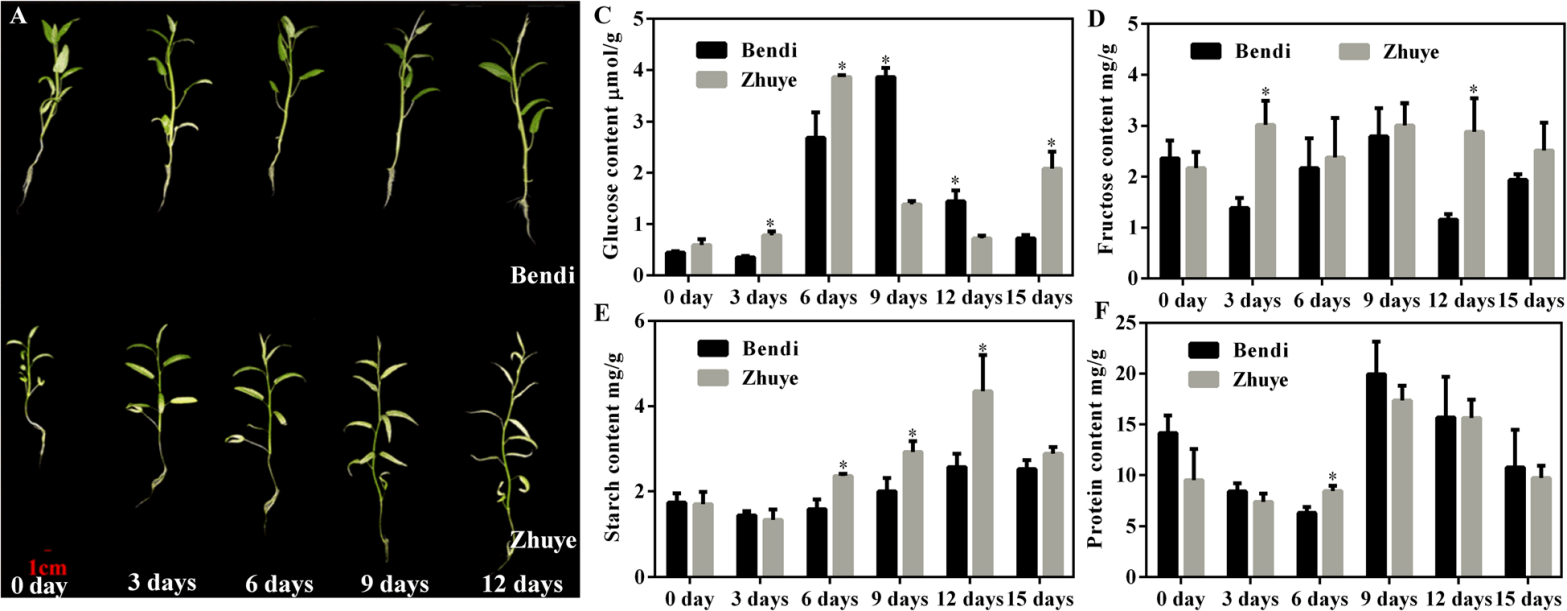
The morphological characterizations of ‘Bendi’ (**a**) and ‘Zhuye’ (**b**) cultivars cultured at 42 °C for 0 day, 3 days, 6 days, 9 days, and 12 days, respectively. Carbohydrate profiles of ‘Bendi’ and ‘Zhuye’ cultivars grown in long-term heat stress. Glucose content (μmol/g) (**c**), fructose content (mg/g) (**d**), starch content (mg/g) (**e**), and protein content (mg/g) (**f**) obtained from ‘Bendi’ and ‘Zhuye’ cultivars cultured at 42 °C for 0 day, 3 days, 6 days, 9 days, 12 days and 15 days, respectively. The data presented are average ± SE of three independent extractions from three replicates. The statistical analyses were conducted in the two varieties at the same time point and the significant one was labelled with asterisks (*)

Transcriptome analysis of water spinach cultivars subjected to long-term heat stress revealed that starch and sucrose metabolism was the main response pathway during heat stress, so as to better understand the effect of high temperature on starch and sucrose metabolism, the contents of glucose, fructose, and starch were tested at different time points (Fig. 7). Obviously, under heat stress condition, cv. ‘Bendi’ showed a much better performance than cv. ‘Zhuye’. Nearly all leaves of cv. ‘Zhuye’ began to yellow after 3-day treatment and to pale when it came to 12 days. For cv. ‘Bendi’, only new leaves showed sensitivity to heat stress (Fig. 7a-b). Carbohydrate of these two cultivars showed changes along with the heat stress (Fig. 7c-f). The content of glucose in cv. ‘Zhuye’ were higher than cv. ‘Bendi’ after treated with high temperature for 3 days. However, the change of every content over time was unique for the two cultivars. For cv. ‘Bendi’, the glucose content showed a peak at 9 day, the fructose content showed a bi-modal pattern with minimum after heat stress for 3 and 12 days, with an increase in between. The starch content was progressively increased and finally stayed stable from 12 days. For cv. ‘Zhuye’, the glucose content showed a bi-modal pattern with a maximum after heat stress for 6 and 15 days, with a decrease in between, the fructose content stayed relatively stable after heat stress, the starch content showed a peak at 12 day. The similar pattern of protein content was found in cvs ‘Bendi’ and ‘Zhuye’, decreased in the first 6 days then peaked at 9 day followed with a continuous decline until 15 days. After treated for 15 days, only glucose content exhibited differential content in cvs ‘Bendi’ and ‘Zhuye’, indicating that glucose metabolism might be one factor affecting the heat resistance behavior in plants.

### DEGs expression analysis and validation with RT-qPCR

**Fig. 8:**
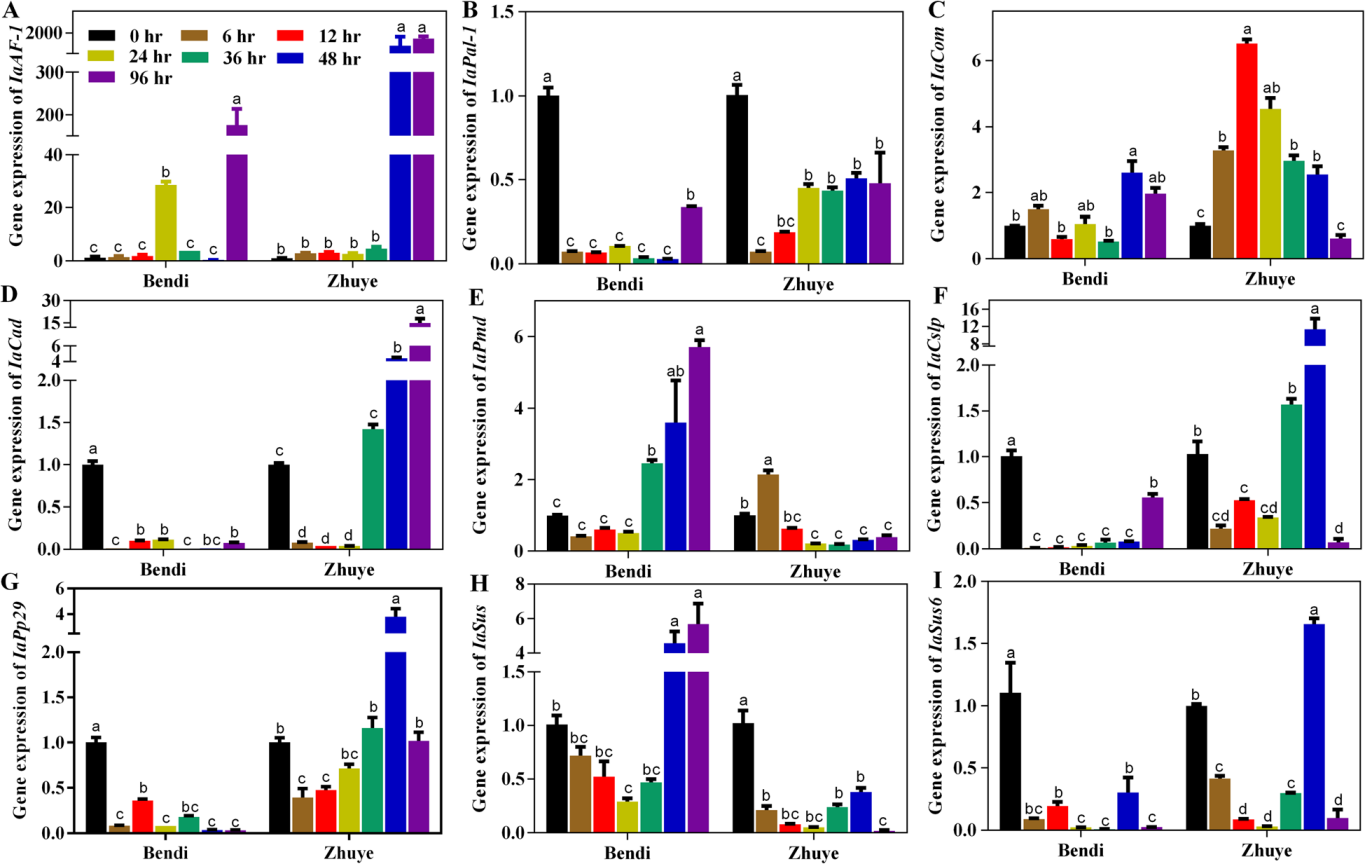
Expression of differentially expressed genes including (**a**) *IaAF-1*, (**b**) *IaPal-1*, (**c**) *IaCom*, (**d**) *IaCad*, (**e**) *IaPmd*, (**f**) *IaCslp*, (**g**) *IaPp29*, (**h**) *IaSus*, and (**i**) *IaSus6* involved in starch and sucrose metabolism and phenylpropanoid biosynthesis during heat stress. Samples were taken from plants before the heat stress treatment (0 h) and after the heat stress treatment (6 h, 12 h, 24 h, 36 h, 48 h and 96 h), respectively. Gene expression was measured by RT-qPCR with the water spinach *U6* gene used as an internal control to normalize the expression data. The bars represent the standard deviation from three independent experiments with three replicates each. The statistical analyses were conducted in the same variety at different time points and different letters were used to indicate the significant differences among samples

To validate the expression data obtained by RNA Seq and investigate the expression patterns of genes involved in starch and sucrose metabolism and phenylpropanoid biosynthesis, a time course (0–96 h) of expression of *IaAF-1*, *IaPal-1*, *IaCom*, *IaCad*, *IaPmd*, *IaCslp*, *IaPp29*, *IaSus*, and *IaSus6* was performed (Fig. 8a-i, Table 1). The expression levels of four genes related to the carbohydrate metabolism (*IaCslp*, *IaPp29*, *IaSus*, and *IaSus6*), four genes related to phenylpropanoid biosynthesis (*IaPal-1*, *IaCom*, *IaCad*, and *IaPmd*) and one gene related to both pathways (*IaAF-1*) varied between cvs ‘Bendi’ and ‘Zhuye’. In the process of heat treatment, expression levels of *AF-1* and *Pp29* in cv. ‘Bendi’ were lower than cv. ‘Zhuye’, and expression levels of *Pal-1* and *Sus* in cv. ‘Bendi’ were higher than cv. ‘Zhuye’, however, other genes showed no particular pattern between cvs ‘Bendi’ and ‘Zhuye’.

**Table 1:** Annotation of RT-qPCR genes in water spinach

Gene name	Gene ID	Swissprot annotation
*IaAF-1*	c37323.graph_c0	Alpha-L-arabinofuranosidase (Precursor)
*IaPal-1*	c17478.graph_c0	Phenylalanine ammonia-lyase
*IaCom*	c7686.graph_c0	Probable caffeoyl-CoA O-methyltransferase At4g26220
*IaCad*	c9393.graph_c0	Probable cinnamyl alcohol dehydrogenase 6
*IaPmd*	c13676.graph_c0	Probable mannitol dehydrogenase
*IaCslp*	c20112.graph_c0	Cellulose synthase-like protein D5
*IaPp29*	c32460.graph_c1	Probable pectinesterase 29 (Precursor)
*IaSus*	c21155.graph_c0	Sucrose synthase
*IaSus6*	c18597.graph_c0	Sucrose synthase 6

The expression level of *AF-1* in both cultivars were low in the early heat treatment while sharp increase in the later times, the expressions level of *AF1* had two peaks at 24 h and 96 h in cv. ‘Bendi’, while increased significantly at 48 h and 96 h in cv. ‘Zhuye’ (Fig. 8a). For genes related to phenylpropanoid biosynthesis, similar decline pattern of *Pal-1* expression was found in cvs ‘Bendi’ and ‘Zhuye’. The *IaCad* in cv. ‘Bendi’ was decreased while in cv. ‘Zhuye’ increased at 36 h and peaked at 96 h. The *IaCom* had fluctuating expression over time and in cv. ‘Bendi’ peaked at 48 h while in cv. ‘Zhuye’ peaked at 12 h. The expression levels of *IaPmd* in cv. ‘Bendi’ declined in the process of heat treatment for 24 h and progressively increased until 96 h and in cv. ‘Zhuye’ it increased at 6 h and subsequently decreased and kept at a low level (Fig. 8b-e). For genes related to carbohydrate metabolism, the expression level of *IaSus* was increased after treated for 48 h and peaked at 96 h in cv. ‘Bendi’ while kept at a low level in cv. ‘Zhuye’ after heat stress. The expression levels of *IaCslp*, *IaPp29* and *IaSus6* were decreased after heat stress in cv. ‘Bnedi’ and peaked at 48 h in cv. ‘Zhuye’ compared with 0 h (Fig. 8f-i). In summary, the response of genes related carbohydrate metabolism and phenylpropanoid biosynthesis differed in cvs ‘Bendi’ and ‘Zhuye’ under heat stress conditions.

## Discussion

Water spinach is a commercially important vegetable distributed in humid areas from subtropical to temperate zones with high tolerance to heat [2, 3]. Understanding the underlying thermotolerant mechanisms of water spinach is essential for improving tolerance to heat stress in plants. However, the physiological and molecular responses to heat stress of water spinach have not been reported and the thermotolerant mechanisms have not been uncovered. In the present study, four water spinach varieties (‘Bendi’, ‘Liuye’, ‘Taiguo’, and ‘Zhuye’) were subjected to prolonged heat stress and showed differential resistance ability to heat (Fig. 1). The reduced photosynthetic efficiency differed among the four cultivars (Fig. 2). The antioxidant capacity was also decreased upon heat stress yet the role of antioxidant level was not predominant in a prolonged thermotolerance since the accumulation pattern of H_2_O_2_ in water spinach was independent of their heat resistance characteristics (Fig. 3). Transcriptomic analysis of heat tolerant and heat sensitive cultivars (Figs. 4, 5 & Fig. 6) indicated that the metabolic pathway such as carbohydrate and phenylpropanoid biosynthesis may play an important role in thermo resistance. Further measurement of carbohydrates (Fig. 7) and the gene expression evaluation (Fig. 8) validated the involvement of sugar transport and metabolic transition in heat resistance.

### High temperature induced inhibition of photosynthesis

High temperature has become an increasingly serious agricultural problem and the heat stress directly impacts carbon utilization and distribution in plants. Studies revealed that the heat stress inhibited chlorophyll content, photosynthesis, carbon fixation, and sucrose metabolism, while increased respiration [19, 20]. Thus, exposure to heat stress would cause a decrease in photosynthetic efficiency [21]. In the present study, prolonged high temperatures treatments decreased chlorophyll contents and Fv/Fm values, while increased F0 values, indicating that the photosynthetic apparatus was damaged after long-term heat stress. Likewise, net photosynthetic rate and transpiration rates in orchardgrass were drastically reduced during heat treatment [22]. However, the chlorophyll content and Fv/Fm decreased in heat-sensitive tomato cultivar upon heat treatment, whereas they were unaltered in heat-tolerant tomato cultivar during heat stress [20]. Chlorophyll a emits fluorescence quickly and change of chlorophyll a related parameter can be used to evaluate the damage to PSII [23–25]. For example, Fv/Fm was regarded as the most sensitive and heat labile component, which primarily limits photochemistry in response to environmental perturbations and stresses [26]. In the present study, the Fv/Fm values in both thermo-tolerant and thermo-sensitive water spinach cultivars were decreased under prolonged heat stress. Beyond photosynthetic indexes, stay-green trait has been developed as an indicator for the identification of heat tolerant plants [27–29]. Stay-green genotypes constituted an important source of germplasm for the genetic improvement of crops with strong resistance to heat [27]. Kumar et al. (2013) revealed that stay-green trait could be used as an effective indicator for tolerance to heat stress in wheat [30]. Emebiri (2013) showed that the maintenance of green color during seed development of barley is closely related to grain productivity and plant resistance to heat [28]. In the present study, the thermo-sensitive cultivar showed much earlier leaf senescence than thermo-tolerant cultivar under heat stress. Furthermore, quantitative trait loci linked to the stay-green trait has been mapped in wheat and sorghum [29–32]. However, heat stress tolerance is a complex trait, and it is difficult to identify one parameter that accurately and consistently predict heat stress tolerance.

### Elevated temperature induced oxidative damage

Plants can be damaged by the accumulation of ROS when they are subjected to long-term abiotic stress conditions [33]. The efficiency of ROS detoxification is closely related to the tolerance of plants to abiotic stresses. In the present study, the polyphenol content and total antioxidant capacity of water spinach were reduced under prolonged heat stress, however, the thermo-tolerant cultivars showed no stronger antioxidant capacity than thermo-sensitive cultivars. Other, thermo-tolerant cultivars also showed no stronger detoxification of ROS than thermo-sensitive cultivars (Fig. 3). These results suggested that prolonged heat stress caused a reduced antioxidant capacity while the role of the antioxidant capacity in prolonged thermotolerance of water spinach was not predominant.

Phenylpropanoids have also been reported as antioxidant agents which comprise a wide and important class of secondary metabolites and have been suggested to play different and significant roles in plant responses to biotic and abiotic stresses [34–36]. Frequently a shift in metabolites of the phenylpropanoid pathway is observed under heat-stress condition. Rienth et al. (2014) revealed that significant differences exist in heat stress responsive pathways of grapevine fruit according to day or night treatment, in particularly regarding genes associated with acidity and phenylpropanoid metabolism [37]. Gao et al. (2017) found that phenylpropanoid biosynthesis pathway was significantly enriched in *Clematis apiifolia* under heat stress [16]. These results were in accordance with our data that phenylpropanoid biosynthesis was also a main pathway which revealed by transcriptome analysis of thermo-tolerant and thermo-sensitive water spinach cultivars under heat stress.

Furthermore, expression of genes related to phenylpropanoid biosynthesis was affected in response to heat stress conditions (Fig. 8). Phenylalanine ammonia-lyase is a key intermediate at the crossroads of the phenolics and lignin synthetic pathways. Especially, the transcript levels of *PAL* differed in thermo-tolerant and -sensitive cultivars. In addition, the caffeic acid 3-O-methyltransferase (Com) encoding gene, involved in the monolignol synthesis, was up-regulated in both cultivars upon heat stress treatment.

### Effect of elevated temperature on sugar metabolism

Heat stress is a complex trait and many genes are involved in various physiological and biochemical processes after heat treatment. Hence, the study of a complete gene expression profile rather than a few genes may provide information about heat-resistant mechanisms in plants. In the present study, RNA-Seq was applied to identify genes and regulatory networks involved in long-term thermotolerance, and enrichment analysis of DEGs showed that the main metabolic differences between thermo-sensitive and thermo-tolerant cultivar were the carbohydrate metabolism and phenylpropanoid biosynthesis.

The heat stress may perturb the carbon balance in a plant due to a low carbohydrate supply caused by the decrease in leaf photosynthetic yield and an increased carbohydrate demand as a result of increased dark respiration and photorespiration [38, 39]. Sugars act as signaling molecules during plant development and it has been observed that leaf senescence is accompanied by high sugar levels [40]. For instance, in *Arabidopsis* leaves, the content of glucose and fructose increased as senescence developed [41]. In rice, large amounts of sugars accumulated in leaves despite a reduction in photosynthetic activity during senescence [42]. In the present study, the results of carbohydrate profiles showed that thermo-sensitive cultivar accumulated more sugar than thermo-tolerant cultivar in the process of prolonged heat stress except for 9 and 12 days, which is in accordance with the above observations.

In the present study, the transcript abundance of 52 genes involved in starch and sucrose metabolism in two cultivars was differentially expressed in response to prolonged heat stress (Table S3 and S4), and the results of RT-qPCR (Fig. 8) also suggested that the heat stress altered sugar assimilation and metabolism and associated changes in transcriptional activity of genes involved in sugar transport and metabolic transition.

## Conclusions

In conclusion, plants respond to heat stress at a given time with complex and integrated mechanisms depending on stress intensity, duration, and plant developmental stage. The prolonged heat stress caused reduced antioxidant capacity while the role of antioxidant capacity in prolonged thermotolerance of water spinach was not predominant. Transcriptome analysis and carbohydrates measurement as well as gene expression evaluation indicated that response of metabolic pathway like carbohydrate and phenylpropanoid biosynthesis to heat stress may be the key player in thermo resistance.

## Methods

### Plant materials and treatments

Four water spinach (*I. aquatica*) cultivars, ‘Bendi’, ‘Zhuye’, ‘Taiguo’, and ‘Liuye’ were used in the study. Seeds of water spinach were produced in Minhuang Seed Industry Co. (Fuzhou, CN) and identified by Joint FAFU-Dalhousie Lab for experimental use. The description of water spinach can be found in HONG KONG Herbarrium with a family No. 283 (https://www.herbarium.gov.hk). They were cultivated in the greenhouse of College of Horticulture in Fujian Agricultural and Forestry University. Among the four cultivars, cvs ‘Bendi’ and ‘Taiguo’ adapted to high temperature which were used in the summer as heat-tolerant cultivars, whereas cvs ‘Zhuye’ and ‘Liuye’ are heat-sensitive cultivars which were used mainly in the winter. Seeds of the four cultivars were germinated in sterile petri dishes (Φ =150 mm) with sterile filter paper at 28 °C. For the phenotypic, photosynthesis, and histochemical analysis of the four varieties, seedlings were transferred to the pots containing peat soil: vermiculite: perlite at 3:1:1 and placed in the chambers at 25 °C or 42 °C. Due to the limited seeds supply of cultivars ‘Taiguo’ and ‘Liuye’, cultivars ‘Bendi’ and ‘Zhuye’ were used in the following experiments to study the heat-resistance mechanism of water spinach. For the further analysis of ‘Bendi’ and ‘Zhuye’, seedlings were transferred to Hoagland solution after germination [43]. Seedlings with six fully expanded leaves were subjected to heat stress treatment of 42 °C for 15 days; seedlings grown at 25 °C were used as control. Then the leaves were used to measure the following parameters of physiological experiments. All measurements were done with three plants and repeated three times independently. Fujian Agriculture and Forestry University owned the land and had approved the study. All these cultivars were sold commercially and no protected species were sampled. No specific permissions were required for these locations/activities.

### Assay of parameters related to photosynthesis in water spinach

The content of photosynthetic pigments, chlorophyll (a and b) contents were estimated by the spectrophotometer following Arnon’s Method (1949) [44], and carotenoid content was determined using a method from Rodriguez-Amaya and Kimura (2004) [45]. The parameters of chlorophyll fluorescence were obtained by using fluorescence monitoring system (Hansatech, Norfolk, UK). After dark adaption for 20 min, the F_0_ and Fm values of leaves was recorded immediately and F′m was measured with the intensity of saturation pulses at 1800 μmol/m^2^/s^1^ for 3 s. The variable fluorescence Fv was calculated as Fm - F_0_ and non-photochemical quenching (NPQ) was expressed as (F′m-Fm)/(F′m).

### Histochemical detection of superoxide (O_2_·^−^) anion and hydrogen peroxide (H_2_O_2_) accumulation

The histochemical detection of O_2_·^−^ and H_2_O_2_ was carried out by using NBT and DAB [46, 47]. First, the water spinach leaves were collected and rinsed with distilled water. Then the leaves were placed in petri dishes and stained by NBT or DAB solution, respectively. The NBT and DAB solution was drained off after overnight staining under darkness at 28 °C. To better visualize the stain, the chlorophyll was removed by soaking leaves in absolute ethanol and boiling in water for 10 min. Finally, leaves with dark blue stain (O_2_·^−^) and reddish-brown stain (H_2_O_2_) were visualized and photographed.

### Analysis of total polyphenol content and antioxidant capacity

The total polyphenol contents in four water spinach cultivars were measured by Folin–Ciocalteu procedure as described before [48]. Samples were ground with methanol and then centrifuged at 4 °C for 20 min at 10,000 rpm. The total polyphenol content was calculated as a catechin equivalent from the calibration curve of catechin standard solutions and expressed as mg catechin/100 g dry plant material. The antioxidant capacity was measured by 2, 2-azobis-3-ethylbenzothiazoline-6-sulfonic acid (ABTS) method [49]. Trolox stock solution was used to perform the calibration curves for antioxidant ability assay. The values were expressed as μmol equivalents of trolox per gram of sample.

### RNA extraction and cDNA library preparation

Leaves of water spinach cultivars ‘Taiguo’ (T01) and ‘Liuye’ (T02) grown at 42 °C for 15 days were collected and total RNAs were extracted by using RNA Extraction Kit (Bioteke Co., Beijing, CN) according to the manufacturer’s instructions. The purity and concentration of the total RNA was detected by NanoDrop 1000 spectrophotometer (Thermo Fisher Scientific, Wilmington, DE, USA) and Qubit® 2.0 Flurometer (Life Technologies, CA, USA), respectively. Two independent paired-end libraries were subjected to RNA Seq analysis. Paired-end libraries with average insert lengths of ~ 250 bp were synthesized by using a Genomic Sample Prep Kit (Illumina, San Diego, CA) according to the manufacturer’s instructions. Before cluster generation, library concentration and size were assayed by using an Agilent DNA 1000 Kit (Agilent, Palo Alto, CA) on a 2100 Bioanalyzer. Libraries were sequenced on an Illumina HiSeq 2500 instrument at Biomarker Technologies Corporation (Beijing, China).

### Transcript assembly and functional annotation

After sequencing, the raw reads with low-quality reads and adaptor sequences were filtered and cleaned. Clean reads were assembled into non-redundant transcripts by using Trinity with a minimum kmer coverage set to 2 and all other parameters were set by default [50]. The resulting sequences were used in BLAST algorithm-based searches and annotations against the National Center for Biotechnology Information non-redundant protein sequences (www.ncbi.nlm.nih.gov/), Kyoto Encyclopedia of Gene and Genomes (KEGG) Ortholog (www.genome.jp/kegg/kaas/) databases, and the manually annotated and curated protein sequence database (Swiss-Prot) (www.expasy.ch/sport) with an *e*-value threshold of 10^− 5^. The functional annotation of Gene Ontology (GO) terms was analyzed by using Blast2go software (www.blast2go.com/). The sequencing data was uploaded to NCBI Sequence Read Archive (PRJNA647931).

### Identification of differentially expressed genes in water spinach

The differentially expressed genes (DEGs) were identified from the FPKM (expected number of Fragments per Kilobase of transcript sequence per Million base pairs sequenced) values using the DEGseq R package [51, 52]. *P* value was adjusted using the q value, and the q value < 0.005 and |log2(fold change) | > 1 was set as the threshold for significantly differential expression [52].

### Analysis of leaf carbohydrate and protein content in water spinach

Samples for carbohydrate analysis were taken from leaves on plants before the heat stress treatment (0 day) and after the heat stress treatment (3, 6, 9, 12, and 15 days). The extraction and determination of glucose, fructose, and starch content were conducted according to the previous method [53]. The protein content was measured by coomassie brilliant blue method and detected at 620 nm using a spectrophotometer.

### Gene expression analysis of DEGs in water spinach

To validate the DEGs from RNA-Seq, Real-Time quantitative PCR (RT-qPCR) analyses were performed. cDNAs were obtained from plants before the heat stress treatment (0 h) and after the heat stress treatments (6 h, 12 h, 24 h, 36 h, 48 h, and 96 h) by using PrimeScript™ RT reagent Kit with gDNA Eraser (Takara, Otsu, JP). RT-qPCR analyses were performed as previously reported [14, 54]. The water spinach gene *U6* was used as an internal control. The primers for RT-qRT-PCR are listed in Table S5.

### Statistical analysis

Statistical analysis was performed by using SPSS (version 19.0, Chicago, IL, USA). To compare the heat resistance related indexes (Figs. 1, 2, 3 & Fig. 7) in the cultivars, Student’s t-tests was used and the significant one was labelled with asterisk (*). The gene expression data at different time points (Fig. 8) was analyzed by one-way analysis of variance (ANOVA) and different letters (e.g. a, b, c, d) were used to indicate the significant differences at *P* < 0.05.

## Supplementary information

**Additional file 1: Supplemental Table 1.** Summary of RNA-Seq data sets. **Supplemental Table 2.** Summary of single nucleotide polymorphisms (SNPs). **Supplemental Table 3.** The DEGs involved in starch and sucrose metabolism. **Supplemental Table 4.** The DEGs involved in phenylpropanoid biosynthesis. **Supplemental Table 5.** Primers used in the verification of differentially expressed genes of heat-tolerant and heat-sensitive water spinach cultivars.

## Data Availability

The datasets generated and/or analyzed during the current study are available in the NCBI Sequence Read Archive repository (PRJNA647931).

## Abbreviations

ABTS: 2-azobis-3-ethylbenzothiazoline-6-sulfonic acid
AF: Alpha-L-arabinofuranosidase
BP: Biological process
Cad: Cinnamyl alcohol dehydrogenase 6
CC: Cellular component
Com: Caffeic acid 3-O-methyltransferase
Cslp: Cellulose synthase-like protein D5
DAB: 3,3′ -diaminobenzidine
DEGs: Differentially expressed genes
FPKM: Fragments per kilobase of transcript sequence per million base pairs sequenced
GO: Gene ontology
H_2_O_2_: Hydrogen peroxide
KEGG: Kyoto encyclopedia of gene and genomes
MF: Molecular function
NBT: Nitro blue tetrazolium
NPQ: Non-photochemical fluorescence quenching
O_2_·^-^: Superoxide
Pmd: Probable mannitol dehydrogenase
PAL: Phenylalanine ammonia-lyase
Pp29: Probable pectinesterase 29
RT-qPCR: Real-Time quantitative PCR
RNA Seq: RNA sequencing
ROS: Reactive oxygen species
SNPs: Single nucleotide polymorphisms
Sus: Sucrose synthase
