# Supplementary material for: Physiological and transcriptomic responses of water spinach (Ipomoea aquatica) to prolonged heat stress
Source: BMC Genomics. 2020 Aug 3;21:533. doi: 10.1186/s12864-020-06953-9 (PMC7430824; doi:10.1186/s12864-020-06953-9)
Supplement: Supplementary file 1 — Additional file 1: Supplemental Table 1. Summary of RNA-Seq data sets. Supplemental Table 2. Summary of single nucleotide polymorphisms (SNPs). Supplemental Table 3. The DEGs involved in starch and sucrose metabolism. Supplemental Table 4. The DEGs involved in phenylpropanoid biosynthesis. Supplemental Table 5. Primers used in the verification of differentially expressed genes of heat-tolerant and heat-sensitive water spinach cultivars. [file 12864_2020_6953_MOESM1_ESM.docx]

**Physiological and transcriptomic responses of water spinach (*Ipomoea aquatica*) to prolonged heat stress**

Rongfang Guo^a, b^, Xingru Wang^a^, Xiaoyun Han^a^, Xiaodong Chen^b^, Gefu Wang-Pruski^a, c *^

^a^ Joint FAFU-Dalhousie Lab, College of Horticulture, Fujian Agriculture and Forestry University, Fuzhou 350002, China

^b^ College of Horticulture, Fujian Agriculture and Forestry University, Fuzhou 350002, China

^c^ Department of Plant, Food, and Environmental Sciences, Faculty of Agriculture, Dalhousie University, Truro, NS B2N 5E3, Canada

Complete list of e-mail addresses for authors:

Rongfang Guo: [guorofa@163.com](mailto:guorofa@163.com), Xingru Wang: 1980919707@qq.com, Xiaoyun Han: 1127575524@qq.com, Xiaodong Chen: [xdchen007@163.com](mailto:xdchen007@163.com), Gefu Wang-Pruski: [Gefu.Wang-Pruski@Dal.Ca](mailto:Gefu.Wang-Pruski@Dal.Ca)

* Corresponding author: Gefu Wang-Pruski at [Gefu.Wang-Pruski@Dal.Ca](mailto:Gefu.Wang-Pruski@Dal.Ca)

**Table S1. Summary of RNA-Seq data sets**

| **Sample name** | **Clean reads** | **Base number** | **Mapped reads** | **Mapped ratio** | **GC content** | **Q20** | **Q30** |
| --- | --- | --- | --- | --- | --- | --- | --- |
| T01 | 23,750,150 | 5,978,228,686 | 20,411,655 | 85.94% | 46.79% | 92.2% | 85.38% |
| T02 | 26,225,685 | 6,602,182,028 | 22,762,781 | 86.8% | 46.55% | 92.18% | 85.48% |

**Table S2. Summary of single nucleotide polymorphisms (SNPs)**

| **Samples name** | **All SNP** | **Homo SNP** | **Hete SNP** |
| --- | --- | --- | --- |
| T01 | 40,063 | 16,005 | 24,058 |
| T02 | 38,836 | 31,324 | 7,512 |

**Table S3. The DEGs involved in starch and sucrose metabolism**

| **Gene ID** | **Swissprot annotation** | **Log2 FC** |
| --- | --- | --- |
| c33217.graph_c0 | Alpha, alpha-trehalose-phosphate synthase [UDP-forming] 5 | 2.18 |
| c10049.graph_c0 | Probable alpha, alpha-trehalose-phosphate synthase [UDP-forming] 11 | -1.98 |
| c27065.graph_c1 | Probable alpha, alpha-trehalose-phosphate synthase [UDP-forming] 9 | -2.00 |
| c14060.graph_c0 | Alpha-1,4 glucan phosphorylase L isozyme, chloroplastic/amyloplastic (Precursor) | 2.41 |
| c28241.graph_c0 | Alpha-glucosidase (Precursor) | 3.11 |
| c11738.graph_c0 | Alpha-glucosidase (Precursor) | 2.74 |
| c33891.graph_c0 | Beta-glucosidase 11 (Precursor) | -1.97 |
| c28428.graph_c0 | Raucaffricine-O-beta-D-glucosidase | -3.38 |
| c23938.graph_c0 | lysosomal beta glucosidase-like | 3.28 |
| c32829.graph_c0 | Alpha-glucan phosphorylase 2, cytosolic | 1.70 |
| c37323.graph_c0 | Alpha-L-arabinofuranosidase (Precursor) | 3.37 |
| c36452.graph_c0 | Alpha-L-arabinofuranosidase (Precursor) | 2.05 |
| c36608.graph_c0 | Alpha-L-arabinofuranosidase (Precursor) | 3.01 |
| c34716.graph_c0 | Alpha-L-arabinofuranosidase (Precursor) | 1.64 |
| c26538.graph_c0 | Alpha-L-arabinofuranosidase (Precursor) | -6.18 |
| c14550.graph_c0 | Alpha-L-arabinofuranosidase (Precursor) | 2.61 |
| c33459.graph_c0 | Beta-amylase 3, chloroplastic (Precursor) | -2.03 |
| c27670.graph_c0 | Beta-amylase 1, chloroplastic (Precursor) | -1.79 |
| c34691.graph_c0 | Probable alpha-amylase 2 | -2.03 |
| c27013.graph_c1 | Beta-D-xylosidase 4 (Precursor) | 2.01 |
| c18031.graph_c0 | Probable beta-D-xylosidase 5 (Precursor) | 5.78 |
| c27665.graph_c1 | Probable beta-D-xylosidase 6 (Precursor) | 1.90 |
| c32334.graph_c0 | Beta-fructofuranosidase, insoluble isoenzyme CWINV1 (Precursor) | 1.99 |
| c32334.graph_c1 | Beta-fructofuranosidase, insoluble isoenzyme CWINV5 (Precursor) | 1.82 |
| c20112.graph_c0 | Cellulose synthase-like protein D5 | 5.09 |
| c14182.graph_c0 | Cellulose synthase-like protein D5 | 4.02 |
| c19579.graph_c0 | Endoglucanase 24 (Precursor) | 2.09 |
| c19332.graph_c0 | Glucose-1-phosphate adenylyltransferase large subunit 1 (Fragment) | -1.97 |
| c37030.graph_c0 | Pectinesterase | 5.10 |
| c34109.graph_c0 | Pectinesterase U1 (Precursor) | 1.83 |
| c21569.graph_c0 | Pectinesterase U1 (Precursor) | 1.85 |
| c32460.graph_c1 | Probable pectinesterase 29 (Precursor) | 5.53 |
| c26094.graph_c0 | Probable pectinesterase 53 (Precursor) | 4.70 |
| c26079.graph_c0 | Pectinesterase 54 (Precursor) | -1.72 |
| c36773.graph_c0 | Probable fructokinase-6, chloroplastic | 1.78 |
| c26581.graph_c0 | Probable polygalacturonase At2g43860 (Precursor) | -3.49 |
| c22886.graph_c0 | Probable sucrose-phosphate synthase 4 | -3.20 |
| c37379.graph_c0 | Probable trehalose-phosphate phosphatase J | 2.07 |
| c36417.graph_c0 | Sucrose synthase | 2.13 |
| c35124.graph_c0 | Sucrose synthase | 2.32 |
| c10027.graph_c0 | Sucrose synthase | -1.61 |
| c21155.graph_c0 | Sucrose synthase | -1.98 |
| c21155.graph_c1 | Sucrose synthase | 2.30 |
| c10337.graph_c1 | Sucrose synthase | -1.84 |
| c18597.graph_c0 | Sucrose synthase 6 | 2.45 |
| c25423.graph_c0 | UDP-glucuronate 4-epimerase 1 | 2.68 |
| c36529.graph_c0 | UDP-glucuronate 4-epimerase 6 | 3.31 |
| c19426.graph_c0 | UDP-glucose 6-dehydrogenase 1 | 3.57 |
| c14520.graph_c0 | UDP-glucose 6-dehydrogenase 4 | 1.53 |
| c8574.graph_c0 | Vicianin hydrolase | 2.84 |
| c15608.graph_c0 | 1,4-alpha-glucan-branching enzyme | 1.69 |
| c27006.graph_c0 | 4-alpha-glucanotransferase, chloroplastic/amyloplastic (Precursor) | 2.09 |

**Table S4. The DEGs involved in phenylpropanoid biosynthesis**

| **Gene ID** | **Swissprot annotation** | **Log2 FC** |
| --- | --- | --- |
| c37323.graph_c0 | Alpha-L-arabinofuranosidase (Precursor) | 3.37 |
| c36452.graph_c0 | Alpha-L-arabinofuranosidase (Precursor) | 2.05 |
| c36608.graph_c0 | Alpha-L-arabinofuranosidase (Precursor) | 3.01 |
| c34716.graph_c0 | Alpha-L-arabinofuranosidase (Precursor) | 1.64 |
| c26538.graph_c0 | Alpha-L-arabinofuranosidase (Precursor) | -6.18 |
| c36466.graph_c0 | Anthocyanidin 3-O-glucosyltransferase 5 | -1.72 |
| c27013.graph_c1 | Beta-D-xylosidase 4 (Precursor) | 2.01 |
| c27665.graph_c1 | Probable beta-D-xylosidase 6 (Precursor) | 1.90 |
| c33891.graph_c0 | Beta-glucosidase 11 (Precursor) | -1.97 |
| c23938.graph_c0 | lysosomal beta glucosidase-like | 3.28 |
| c28428.graph_c0 | Raucaffricine-O-beta-D-glucosidase | -3.38 |
| c24608.graph_c0 | 8-hydroxygeraniol dehydrogenase | 1.93 |
| c37490.graph_c0 | Caffeic acid 3-O-methyltransferase | 1.78 |
| c19719.graph_c0 | Cinnamoyl-CoA reductase 1 | -1.50 |
| c19719.graph_c1 | Cinnamoyl-CoA reductase 2 | -1.66 |
| c31083.graph_c0 | Peroxidase 3 (Precursor) | 2.60 |
| c28809.graph_c0 | Peroxidase 3 (Precursor) | 1.65 |
| c33543.graph_c0 | Peroxidase 4 (Precursor) | -3.38 |
| c22264.graph_c0 | Peroxidase 4 | -2.60 |
| c19236.graph_c0 | Peroxidase 10 (Precursor) | -3.97 |
| c29866.graph_c0 | Peroxidase 19 (Precursor) | 1.73 |
| c33865.graph_c0 | Peroxidase 21 (Precursor) | -2.82 |
| c11130.graph_c0 | Peroxidase 29 (Precursor) | 2.76 |
| c35582.graph_c0 | Peroxidase 31 (Precursor) | 2.17 |
| c8980.graph_c0 | Peroxidase 42 (Precursor) | 2.71 |
| c34156.graph_c0 | Peroxidase 51 (Precursor) | -1.66 |
| c23327.graph_c0 | Peroxidase 64 (Precursor) | 6.13 |
| c36659.graph_c0 | Peroxidase N1 (Precursor) | -3.62 |
| c27837.graph_c0 | Anionic peroxidase (Precursor) | -1.79 |
| c33681.graph_c0 | Anionic peroxidase (Precursor) | -3.85 |
| c24344.graph_c0 | Cationic peroxidase 1 (Precursor) | -2.74 |
| c8050.graph_c0 | Neutral peroxidase (Precursor) | 2.19 |
| c17478.graph_c0 | Phenylalanine ammonia-lyase | 2.14 |
| c8330.graph_c0 | Phenylalanine ammonia-lyase | 2.58 |
| c7686.graph_c0 | Probable caffeoyl-CoA O-methyltransferase At4g26220 | -2.31 |
| c9393.graph_c0 | Probable cinnamyl alcohol dehydrogenase 6 | 3.56 |
| c13676.graph_c0 | Probable mannitol dehydrogenase | -2.66 |
| c8574.graph_c0 | Vicianin hydrolase (Precursor; Fragment) | 2.84 |
| c27980.graph_c0 | 4-coumarate--CoA ligase 1 | -2.06 |
| c34364.graph_c0 | 4-coumarate--CoA ligase 2 | -1.53 |

**Table S5. Primers used in the verification of differential expressed genes of heat heat-tolerant and heat-sensitive water spinach cultivars.**

| gene name | Sequence (5’-3’) |
| --- | --- |
| *IaAF-1-F* | GGGAGCAAAAACATAAGGGAGTC |
| *IaAF-1-R* | ATGGTAAATGAGATGCAGAAAGGTG |
| *IaPal-1-F* | CGGAATCTTCGGAAATGGAGG |
| *IaPal-1-R* | GGCGATGTAGGATAGCGGGAC |
| *IaCad-F* | TGGAGTCAAAGGGGAAAAGAGTTG |
| *IaCad-R* | GCATGTGTTTAGGGTTGGTGCTAAG |
| *IaPmd-F* | GCAGGTGGAGTAGTGAGGGTGTTG |
| *IaPmd-R* | GAAGGATGGTGGCAGGGAGTTG |
| *IaCslpD5-F* | AGATGAGACAGCACCCAGAAGC |
| *IaCslpD5-R* | GGTCATAGCAGGAGTGGACATAAAC |
| *IaPp29-F* | ATTAAATCCAGCACTCTACCTCTCG |
| *IaPp29-F* | TGGGATGTAAAAGTTGGGCTTG |
| *IaSus-F* | AACTCGGAGAAAATCAAGTAGGGG |
| *IaSus-R* | AGGAAGAGCTTGTGAATGGAAATG |
| *IaSus6-F* | CGGCGATACCTTGAGCTATTCTAC |
| *IaSus6-R* | CTGTCAGTATTGCCTCCACCTTT |
